# Association between HLA Variations and Chronic Hepatitis B Virus Infection in Saudi Arabian Patients

**DOI:** 10.1371/journal.pone.0080445

**Published:** 2014-01-22

**Authors:** Ahmed A. Al-Qahtani, Mashael R. Al-Anazi, Ayman A. Abdo, Faisal M. Sanai, Waleed Al-Hamoudi, Khalid A. Alswat, Hamad I. Al-Ashgar, Nisreen Z. Khalaf, Abdelmoneim M. Eldali, Nisha A. Viswan, Mohammed N. Al-Ahdal

**Affiliations:** 1 Department of Infection and Immunity, Research Center, King Faisal Specialist Hospital & Research Center, Riyadh, Saudi Arabia; 2 Section of Gastroenterology, Department of Medicine, College of Medicine, King Saud University, Riyadh, Saudi Arabia; 3 Hepatobiliary Sciences and Liver Transplantation, King Abdulaziz Medical City, Riyadh, Saudi Arabia; 4 Department of Medicine, King Faisal Specialist Hospital & Research Center, Riyadh, Saudi Arabia; 5 Department of Biostatistics, Epidemiology & Scientific Computing, King Faisal Specialist Hospital & Research Center, Riyadh, Saudi Arabia; 6 Liver Disease Research Center, King Saud University, Riyadh, Saudi Arabia; Yonsei University College of Medicine, Republic of Korea

## Abstract

Hepatitis B virus (HBV) infection is a leading cause of liver diseases including cirrhosis and hepatocellular carcinoma. Human leukocyte antigens (HLAs) play an important role in the regulation of immune response against infectious organisms, including HBV. Recently, several genome-wide association (GWAS) studies have shown that genetic variations in HLA genes influence disease progression in HBV infection. The aim of this study was to investigate the role of HLA genetic polymorphisms and their possible role in HBV infection in Saudi Arabian patients. Variations in HLA genes were screened in 1672 subjects who were divided according to their clinical status into six categories as follows; clearance group, inactive carriers, active carriers, cirrhosis, hepatocellular carcinoma (HCC) patients and uninfected healthy controls. Three single nucleotide polymorphisms (SNPs) belonged to HLA-DQ region (rs2856718, rs7453920 and rs9275572) and two SNPs belonged to HLA-DP (rs3077 and rs9277535) were studied. The SNPs were genotyped by PCR-based DNA sequencing (rs2856718) and allele specific TaqMan genotyping assays (rs3077, rs7453920, rs9277535 and rs9275572). The results showed that rs2856718, rs3077, rs9277535 and rs9275572 were associated with HBV infection (p = 0.0003, OR = 1.351, CI = 1.147–1.591; p = 0.041, OR = 1.20, CI = 1.007–1.43; p = 0.045, OR = 1.198, CI = 1.004–1.43 and p = 0.0018, OR = 0.776, CI = 0.662–0.910, respectively). However, allele frequency of rs2856718, rs7453920 and rs9275572 were found more in chronically infected patients when compared to clearance group infection (p = 0.0001, OR = 1.462, CI = 1.204–1.776; p = 0.0178, OR = 1.267, CI = 1.042–1.540 and p = 0.010, OR = 0.776, CI = 0.639–0.942, respectively). No association was found when polymorphisms in HLA genes were compared in active carriers versus cirrhosis/HCC patients. In conclusion, these results suggest that variations in HLA genes could affect susceptibility to and clearance of HBV infection in Saudi Arabian patients.

## Introduction

Hepatitis B infection is an inflammatory illness of the liver caused by hepatitis B virus (HBV). It is a potentially severe disease accounting for over 400 million chronic HBV patients and nearly 1.2 million deaths every year [1]. Even though 2–10% of HBV-infected individuals develop chronic complications, the clinical outcomes vary, with 15–40% of these chronic HBV patients are at a higher risk of developing liver cirrhosis and hepatocellular carcinoma (HCC) during their lifetime [2]. Although, the exact mechanism is not fully understood, the reason for this difference in response to HBV virus is believed to be attributed to a complex web of inter-related factors, such as host genetic, viral, and environmental factors [3].

Since the outcome of any infection depends mainly on the host immune response, a number of studies have investigated and reported that several variations in the human leukocyte antigens (HLAs) class I and class II genes are involved in HBV persistence or clearance [4], [5], [6]. HLAs belong to the major histocompatibility complex (MHC) genes that are located on chromosome 6p21. MHC class II molecules play an important role in the defense against infections and are involved in presenting antigen to CD4^+^ T cells thereby augmenting antibody production and cytotoxic T cell activation. Such molecules are encoded by three different loci namely HLA-DR, -DQ, and -DP. These genes are highly polymorphic; thus enabling them to present a wide range of antigens [7], [8], [9].

HLA-DP and HLA-DQ are heterodimeric molecules consisting of alpha and beta chains that are encoded by HLA-DPA1, -DQA1 and HLA-DPB1, -DQB1 genes respectively. HLA- DPs and DQs are highly polymorphic especially in exon 2, which codes for antigen-binding sites and, therefore, a number of alleles have been reported to be associated either in persistence of HBV infection including HLA-DQA1*0302 [10], -DQB1*0301 [11], -DQA1*0501 [12], -DPA1*0202 [13] and –DPB1*0501 [13] or in HBV resistance to treatment including HLA-DQB1*0501 and -DQB1*0604 [14].

A recent genome wide association (GWAS) study [13] identified 11 single nucleotide polymorphisms (SNPs) belonging to the class II HLA-DP region to be associated with chronic hepatitis B infection among Japanese subjects. However, upon validation among two independent Japanese and a Thai cohort, it was revealed that only two SNPs (rs3077 and rs9277535) continued to remain significant. A second GWAS study conducted by the same group among Japanese subjects revealed two SNPs (rs2856718 and rs7453920) within the HLA-DQ locus to be significantly associated with hepatitis B persistence [14]. In addition, a study conducted on Chinese population revealed that the non-risk alleles of HLA-DP SNPs, rs3077 and rs9277535, showed protective effects for the clearance of the virus [15]. Similarly, several other studies conducted on different Chinese sub-populations have investigated the role of HLA-DP variants on development of persistent chronic HBV infection or its clearance [16], [17], [18], [19]. Thus, this study aims to determine if similar observations can be monitored when these SNPs are examined in HBV-infected or HBV-cleared individuals of Saudi Arabian origin. Five SNPs were analyzed, two belonging to the HLA-DP region (rs3077 and rs9277535) and three belonging to the HLA-DQ region (rs2856718, rs7453920 and rs9275572).

## Patients and Methods

### Patients

The study protocol conformed to the 1975 Declaration of Helsinki and was approved by the institutional review boards of King Faisal Specialist Hospital and Research Center, Armed Forces Hospital, and King Khalid University Hospital, Riyadh, Saudi Arabia. A total of 1672 Saudi nationals were included in the study who were recruited during a three year period from August 2007 to August 2010. They consisted of the complete spectrum of HBV infected individuals, 488 inactive asymptomatic HBV-carriers (Group I), 208 active symptomatic HBV-carriers (Group II), 85 HBV-infected patients diagnosed with liver cirrhosis or cirrhosis+HCC (Group III) and 304 HBV-cleared subjects (Group IV). The study also included 587 healthy control subjects who were blood donors and were HBs antigen (HBsAg) and HBe antigen (HBeAg) negative. All patients had to sign an informed consent prior to enrolling in the study, and their basic demographic data were recorded. Subjects who were found to be positive for HBsAg and negative for HBeAg with persistently normal serum ALT levels and HBV DNA level <2000 IU/mL were characterized as inactive carriers, while subjects who were found to have a repeated detection of HBsAg over a period of six months and with elevated serum ALT levels and HBV DNA level ≥2000 IU/mL were diagnosed as active HBV carriers. The clearance group was identified as individuals who were diagnosed as anti-HBcore antibody positive and HBsAg and HBeAg negative. Liver cirrhosis among HBV infected patients was confirmed by liver biopsy, clinical, biochemical or radiological evidence of cirrhosis. Diagnosis of HCC was made by computed tomography and/or magnetic resonance imaging of the liver, according to the guidelines published for the diagnosis and management of HCC [20]. Baseline characteristics including age, gender, and clinical data such as biochemical tests and viral load are shown in Table 1.

**Table 1:** Baseline characteristics of all subjects included in this study.

Variable	Inactive	Active	Cirrhosis+HCC	Clearance	Control	p-value
	(n = 488)	(n = 208)	(n = 85)	(n = 304)	(587)	
**Age (yrs)** *	41 (32–54)	37 (25–48)	51 (40–69)	37 (28–45)	29 (24–36)	<0.0001
**Gender**						
Male count (%)	339 (69.46)	158 (75.96)	71 (83.53)	301 (99.01)	554 (94.38%)	<0.0001
Female count (%)	149 (30.54)	50 (24.04)	14 (16.47)	3 (0.99)	33 (5.62%)	
**BMI** *	32.7 (22.3–36.3)	28.7 (21.3–33)	22.8 (19.79–31)			<0.0001
**ALT (IU/L)** §	41.9±124.3	80.3±78.1	83.1±99.7	NA	NA	
**AST (IU/L)** §	51 (34–82)	125 (24–1358)	117 (39–1672)	NA	NA	<0.001
**Viral load** (copies/mL)*	289 (47.0–2173)	1.04×16^6^ (6.9×10^4^–1.0×10^7^)	9832 (601–4.7×10^6^)	NA	NA	<0.0001

NA, Not available; BMI, body-mass index; ALT, alanine aminotransferase; AST, aspartate aminotransferase.

* Values are expressed as median (interquartile ranges),

§ Mean ± SD.

### Genotyping of HLA SNPs

Genomic DNA from peripheral blood mononuclear cells was extracted using Gentra Pure Gene kit according to the manufacturer's protocol (Qiagen, Hilden, Germany). Blood samples from patients and controls were genotyped for the five HLA SNPs using either a) PCR-based genotyping assay or b) TaqMan assay.

- *PCR-based genotyping assay*: Specific primers (forward, 5′-TGACATCAAAACATTTCAACGA-3′; reverse, 5′-CTGCCATCATGACTTCAAGC-3′) for HLADQ-rs2856718 were designed using Primer3v.0.4.0 (http://frodo.wi.mit.edu/primer3). All PCR reactions were performed using the Veriti 96-Well Thermal Cycler (Applied Biosystems), under the following conditions: 2-min initial denaturation at 94°C, followed by 40 cycles of 94°C for 1-min, 56°C for 45 s, 72°C for 1-min and a 5-min final extension at 72°C.
  DNA sequencing: The amplified PCR products were electrophoresed in a 2% agarose gel and visualized using ethidium bromide staining (0.5 µg/ml). The DNA fragments were then analyzed by direct sequencing using BigDye Terminator v3.1 Cycle Sequencing Kit according to the manufacturer's instructions (BigDye® Terminator v3.1 Cycle Sequencing Kit, Applera). A 10 µl reaction mix containing 5 µl PCR products, 2 µl terminator ready reaction mix, 2 µl sequencing buffer, 0.2 µM each primer (either forward or reverse primer specific for the target sequences). The reaction was performed in cycling at 96°C for 1 minute and then at 96°C for 10 seconds, 55°C for 5 seconds and 60°C for 4 minutes for 25 cycles. Sequencing products were purified using DyeEx spin column and eluted in 25 µl ddH2O. Each sample was then vacuum-dried and resuspended in 15 µl of Hi-Di formamide. The samples were analyzed by ABI 3700 DNA Analyzer (Applied Biosystems, USA).
- *TaqMan genotyping assay*: Four HLA SNPs- rs3077, rs9277535, rs9275572 and rs7453920 were genotyped using TaqMan allelic discrimination assay with the 7900 HT Fast Real Time PCR System (Applied Biosystems, Foster City, CA, USA). The amplifying primers and probes were ordered for TaqMan (Applied Biosystems, Foster City, CA, USA). One of the allelic probes was labeled with FAM dye and the other with the fluorescent VIC dye. PCR was run in the TaqMan universal master mix (Applied Biosystems) at a probe concentration of 20×. The reaction was performed in a 96-well format using 20 ng of genomic DNA in a total reaction volume of 25 µl. The reaction plates were heated for 2 mins at 50°C and for 10 mins at 95°C, followed by 40 cycles of 95°C for 15 s and 60°C for 1.5 mins. The fluorescence intensity of each well in TaqMan assay plate was read. Fluorescence data files from each plate were analyzed by automated software (SDS 2.4).

### Statistical analysis

Statistical analysis was performed using SPSS version 17.0 (SPSS Inc., Chicago, IL, USA). The genotypic and allelic distribution for the HLA SNPs among the patient groups, controls and clearance group were assessed by means of Pearsons's χ^2^ test and the association between the SNPs and the disease status were calculated under additive, dominant and recessive genetic models and were expressed in terms of odds ratio (OR) and their 95% confidence intervals (CI). A p≤0.05 was considered to be statistically significant. The SNPs were tested for Hardy–Weinberg equilibrium (HWE) using the DeFinetti program (http://ihg.gsf.de/cgi-bin/hw/hwa1.pl). A cut-off p-value of 0.01 was set for HWE.

## Results

In the present case-control study, two SNPs belonging to the HLA-DP (rs3077 and rs9277535) and three SNPs in HLA-DQ (rs2856718, rs7453920 and rs9275572) were analyzed. The study included 488 inactive HBV carriers, 208 active HBV carriers, 85 HBV-infected patients suffering from cirrhosis and cirrhosis+HCC, 304 HBV-cleared individuals and 587 healthy uninfected controls. The Hardy–Weinberg equilibrium (HWE) was assessed for the five SNPs using the chi-square test with one degree of freedom, however, only rs2856718 deviated from HWE when analyzed for the whole population. This SNP was found to be in HWE in healthy control subjects and therefore none of the SNPs were excluded from the analysis.

The genotypic distribution of the five SNPs among patients and controls are as shown in Table 2. The SNPs rs2856718 (OR = 1.351; 95% C.I. 1.147–1.591; p = 0.0003), rs3077 (OR = 1.200; 95% C.I. 1.007–1.431; p = 0.041) and rs9277535 (OR = 1.198; 95% C.I. 1.004–1.430; p = 0.045) were found to be significantly associated with hepatitis B infection when the HBV-infected patients group was compared against the control group (Table 2). In addition, these three SNPs were found to be recessively associated with the susceptibility to HBV infection, with odds-ratios of 0.513, 1.581 and 1.871, respectively. For HLA-DQ SNPs, no significant association was observed in the case of rs7453920 with regard to HBV susceptibility, the minor allele A of HLA-DQ rs9275572 was found to be recessively associated with HBV infection in the opposite direction with an OR of 0.776, 95% C.I. 0.427–0.821, and p-value of 0.001, suggesting a plausible protective role for homozygous AA genotype against HBV infection.

**Table 2:** Genotypic distribution for HLA gene polymorphisms when patients (Groups I+II+III+IV) were compared to control group.

SNPs	Genotype/Allele distribution	Controls	Patients	OR (95% C.I.)	χ^2^	P-value
**rs2856718**				1.351 (1.147–1.591)	13.02	**0.0003**
	AA	127 (25%)	268 (40%)			
	AG	223 (44%)	205 (30%)			
	GG	155 (31%)	204 (30%)			
	**A**	477 (47%)	741 (55%)			
	G	533 (53%)	613 (45%)			
	AA+AG.vs.GG			1.027(0.799–1.319)	0.04	0.8359
	AA vs. AG+GG			0.513 (0.398–0.661)	27.1	**1.93×10^−7^**
**rs7453920**				1.104 (0.945–1.291)	1.56	0.2118
	AA	90 (15.5%)	144 (18.8%)			
	AG	269 (46.2%)	341 (44.4%)			
	GG	223 (38.3%)	283 (36.8%)			
	**A**	449 (39%)	629 (41%)			
	G	715 (61%)	907 (59%)			
	AA+AG vs. GG			1.065(0.852–1.330)	0.3	0.5813
	AA vs. AG+GG			1.262 (0.936–1.701)	2.5	0.114
**rs3077**				1.200 (1.007–1.431)	4.16	**0.0414**
	GG	34 (6%)	69 (9%)			
	AG	206 (35%)	279 (36%)			
	AA	347 (59%)	431 (55%)			
	**G**	274 (23%)	417 (27%)			
	A	900 (77%)	1141 (73%)			
	GG+AG vs. AA			1.167 (0.940–1.450)	1.96	0.1617
	GG vs. AG+AA			1.581 (1.013–2.474)	4.51	**0.034**
**rs9277535**				1.198 (1.004–1.430)	4.01	**0.0453**
	GG	27 (5%)	65 (8.5%)			
	AG	216 (38%)	284 (37.1%)			
	AA	328 (57%)	416 (54.4%)			
	**G**	270 (24%)	414 (27%)			
	A	872 (76%)	1116 (73%)			
	GG+AG vs. AA			1.132 (0.910–1.409)	1.24	0.2647
	GG vs. AG+AA			1.871 (1.152–3.052)	7.24	**0.007**
**rs9275572**				0.776 (0.662–0.910)	9.75	**0.0018**
	AA	97 (17.0%)	84 (10.8%)			
	AG	251 (44.0%)	347 (44.7%)			
	GG	223 (39.1%)	346 (44.5%)			
	**A**	445 (39.0%)	515 (33.1%)			
	G	697 (61.0%)	1039 (66.9%)			
	AA+AG vs. GG			0.798 (0.641–0.994)	4.05	**0.0443**
	AA vs. AG+GG			0.592 (0.427–0.821)	10.8	**0.001**

Risk alleles marked in **BOLD** letters.

When these SNPs were analyzed to determine whether they play a role in clearing the HBV virus, SNPs rs2856718 (OR = 1.462; 95% C.I. 1.204–1.776; p = 0.0001), rs7453920 (OR = 1.267; 95% C.I. 1.042–1.540; p = 0.017), and rs9275572 (OR = 0.776; 95% C.I. 0.639–0.942; p = 0.0104) were found to have a significant association (Table 3). The frequency of rs2856718-G allele of among HBV-cleared individuals (freq. = 0.55) was higher than that of HBV-infected patients (freq. = 0.45), and the G allele was found to be dominantly associated with an OR of 3.640 (95% C.I. 2.523–5.265) and a p<0.0001, suggesting that the G allele may have an important role in clearing the HBV virus. Similarly, the rs7453920-G was found to be dominantly associated with HBV clearance with an OR of 1.812 (95% C.I. 1.194–2.761) and p = 0.0030. Also, the frequency of rs9275572-A was found to be more in HBV-infected than those who cleared the virus.

**Table 3:** Genotypic distribution for HLA gene polymorphisms when patients (Groups I+II+III+IV) were compared to clearance group.

SNPs	Genotype/Allele distribution	Clearance	Patients	OR (95% C.I.)	χ^2^	P-value
**rs2856718**				1.462 (1.204–1.776)	14.77	**0.0001**
	AA	45 (15%)	268 (40%)			
	AG	177 (60%)	205 (30%)			
	GG	73 (25%)	204 (30%)			
	**A**	267 (45%)	741 (55%)			
	G	323 (55%)	613 (45%)			
	AA+AG.vs.GG			0.762(0.559–1.041)	2.93	0.0871
	AA vs. AG+GG			3.640 (2.523–5.265)	55.72	**<0.0001**
**rs7453920**				1.267 (1.042–1.540)	5.62	**0.0178**
	AA	34 (11%)	144 (18.8%)			
	AG	145 (48%)	341 (44.4%)			
	GG	122 (41%)	283 (36.8%)			
	**A**	213 (35.4%)	629 (41%)			
	G	389 (64.6%)	907 (59%)			
	AA+AG vs. GG			1.168 (0.889–1.534)	1.25	0.2643
	AA vs. AG+GG			1.812 (1.194–2.761)	8.66	**0.003**
**rs3077**				1.116 (0.899–1.386)	0.99	0.3205
	GG	24 (8%)	69 (9%)			
	AG	100 (33%)	279 (36%)			
	AA	176 (59%)	431 (55%)			
	**G**	148 (25%)	417 (27%)			
	A	452 (75%)	1141 (73%)			
	GG+AG vs. AA			1.146 (0.875–1.501)	0.98	0.3218
	GG vs. AG+AA			1.118 (0.672–1.870)	0.2	0.653
**rs9277535**				1.021 (0.825–1.263)	0.04	0.85
	GG	24 (8%)	65 (8.5%)			
	AG	113 (37%)	284 (37.1%)			
	AA	165 (55%)	416 (54.4%)			
	**G**	161 (27%)	414 (27%)			
	A	443 (73%)	1116 (73%)			
	GG+AG vs. AA			1.010 (0.773–1.320)	0.01	0.9396
	GG vs. AG+AA			1.076 (0.644–1.806)	0.09	0.77
**rs9275572**				0.776 (0.639–0.942)	6.57	**0.0104**
	AA	48 (16%)	84 (10.8%)			
	AG	141 (46%)	347 (44.7%)			
	GG	115 (38%)	346 (44.5%)			
	**A**	237 (39%)	515 (33.1%)			
	G	371 (61%)	1039 (66.9%)			
	AA+AG vs. GG			0.758 (0.578–0.994)	4.01	**0.0452**
	AA vs. AG+GG			0.646 (0.434–0.965)	5.052	**0.025**

Risk alleles marked in **BOLD** letters.

On comparing groups II, III and IV with inactive HBV carriers (group I), none of the SNPs were found to have a significant association with HBV persistence (Table 4). However, rs3077-G allele was found to be dominantly associated with HBV persistence but in the negative direction (OR = 0.675, 95% C.I. 0.502–0.908; p = 0.0092).

**Table 4:** Genotypic distribution for HLA gene polymorphisms when groups II+III+IV were compared to group I.

SNPs	Genotype/Allele distribution	Inactive	Active+Cirr+HCC	OR (95% C.I.)	χ^2^	P-value
rs2856718				1.050(0.841–1.313)	0.19	0.6649
	AA	169 (39.6%)	99 (40%)			
	AG	127 (29.7%)	77 (31%)			
	GG	131 (30.7%)	71 (29%)			
	**A**	465 (54%)	275 (56%)			
	G	389 (46%)	219 (44%)			
	AA+AG.vs.GG			1.097(0.778–1.547)	0.28	0.5974
rs7453920				1.027 (0.832–1.269)	0.06	0.8023
	AA	90 (18.6%)	54 (19.2%)			
	AG	215 (44.4%)	125 (44.5%)			
	GG	179 (37%)	102 (36.3%)			
	**A**	395 (41%)	233 (41.5%)			
	G	573 (59%)	329 (58.5%)			
	AA+AG vs. GG			1.030(0.759–1.398)	0.04	0.8498
rs3077				0.799(0.631–1.012)	3.46	0.0630
	GG	41 (8.4%)	27 (9%)			
	AG	194 (39.8%)	84 (29%)			
	AA	253 (51.8%)	177 (62%)			
	**G**	276 (28%)	138 (24%)			
	A	700 (72%)	438 (76%)			
	GG+AG vs. AA			0.675 (0.502–0.908)	6.78	**0.0092**
rs9277535				0.831 (0.656–1.053)	2.35	0.1253
	GG	45 (9%)	20 (7%)			
	AG	183 (38%)	100 (36%)			
	AA	252 (53%)	162 (57%)			
	**G**	273 (28%)	140 (25%)			
	A	687 (72%)	424 (75%)			
	GG+AG vs. AA			0.819(0.609–1.101)	1.75	0.1856
rs9275572				1.047 (0.842–1.303)	0.17	0.6796
	AA	51 (10.5%)	33 (11.4%)			
	AG	216 (44.4%)	129 (44.6%)			
	GG	219 (45.1%)	127 (43.9%)			
	**A**	318 (32.7%)	195 (33.7%)			
	G	654 (67.3%)	383 (66.3%)			
	AA+AG vs. GG			1.046 (0.780–1.403)	0.09	0.7623

Risk alleles marked in **BOLD** letters.

Similarly, no significant association was observed with regards to the development of Cirrhosis+HCC among HBV-infected patients when groups III+IV were compared to group II (active HBV carriers) (Table 5).

**Table 5:** Genotypic distribution for HLA gene polymorphisms when groups III+IV were compared to group II.

SNPs	Genotype/Allele distribution	Active	Cirr+HCC	OR (95% C.I.)	χ^2^	P-value
**rs2856718**				0.851 (0.575–1.258)	0.66	0.4178
	AA	77 (44%)	22 (31%)			
	AG	46 (26%)	31 (43.6%)			
	GG	53 (30%)	18 (25.4%)			
	**A**	200 (57%)	75 (53%)			
	G	152 (43%)	67 (47%)			
	AA+AG.vs.GG			1.269 (0.680–2.368)	0.56	0.4543
**rs7453920**				1.320(0.914–1.908)	2.19	0.1385
	AA	35 (17.5%)	19 (23.4%)			
	AG	88 (44%)	37 (45.7%)			
	GG	77 (38.5%)	25 (30.9%)			
	**A**	158 (39.5%)	75 (46.3%)			
	G	242 (60.5%)	87 (53.7%)			
	AA+AG vs. GG			1.402(0.808–2.432)	1.45	0.2279
**rs3077**				1.323(0.879–1.991)	1.80	0.1796
	GG	17 (8.4%)	10 (11.8%)			
	AG	57 (28.1%)	27 (31.8%)			
	AA	129 (63.5%)	48 (56.4%)			
	**G**	91 (22%)	47 (28%)			
	A	315 (78%)	123 (72%)			
	GG+AG vs. AA			1.344 (0.803–2.250)	1.27	0.2604
**rs9277535**				1.304 (0.861–1.974)	1.57	0.2096
	GG	12 (6%)	8 (10.1%)			
	AG	71 (34.9%)	29 (36.7%)			
	AA	120 (59.1%)	42 (53.2%)			
	**G**	95 (23.4%)	45 (28.5%)			
	A	311 (76.6%)	113 (71.5%)			
	GG+AG vs. AA			1.274 (0.755–2.149)	0.82	0.3642
**rs9275572**				0.834 (0.565–1.232)	0.83	0.3620
	AA	25 (12.0%)	8 (9.9%)			
	AG	95 (45.7%)	34 (42.0%)			
	GG	88 (42.3%)	39 (48.1%)			
	**A**	145 (34.9%)	50 (30.9%)			
	G	271 (65.1%)	112 (69.1%)			
	AA+AG vs. GG			0.790 (0.472–1.322)	0.81	0.3689

Risk alleles marked in **BOLD** letters.

**Figure 1:**
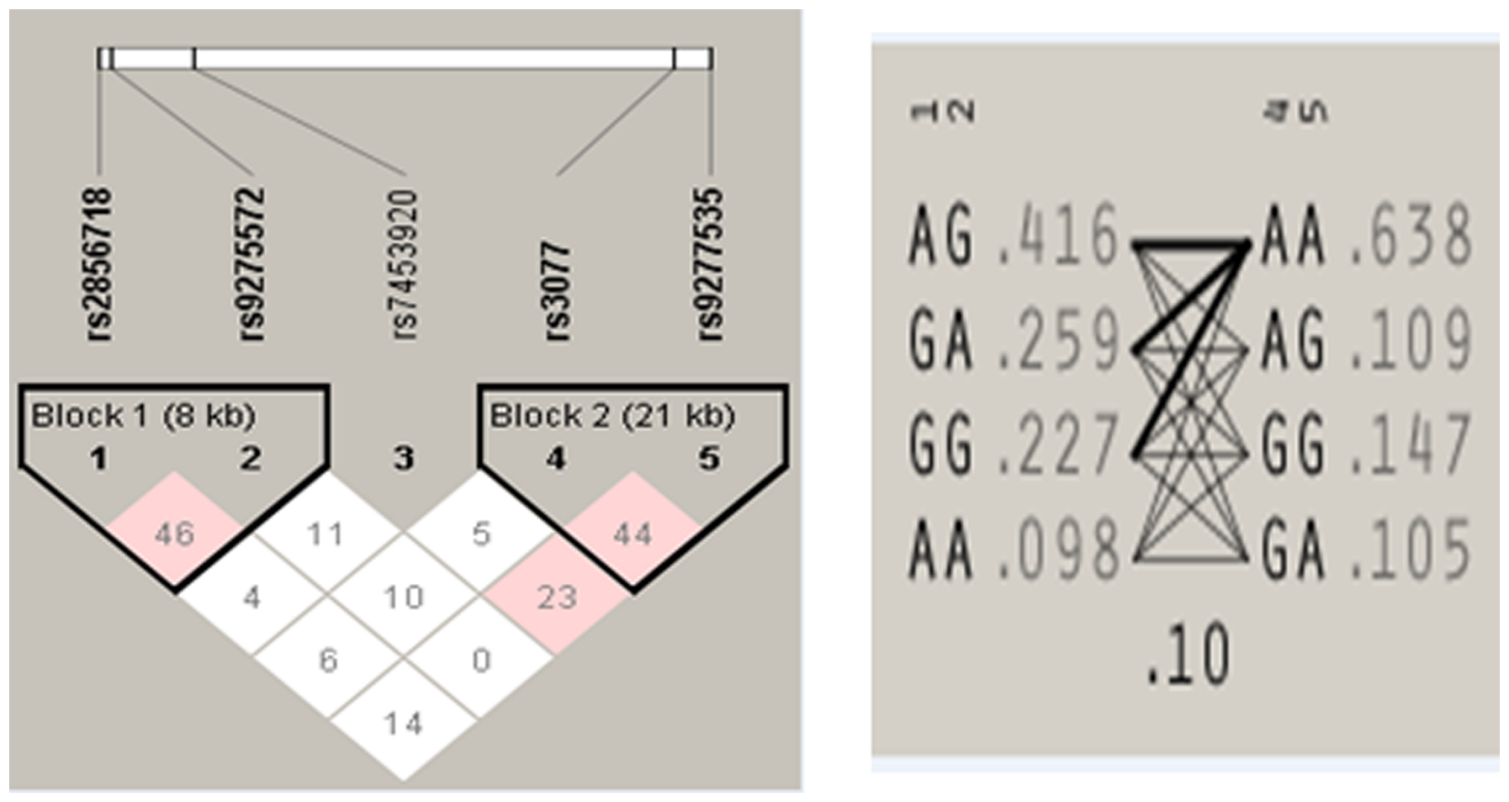
Pair-wise linkage disequilibrium analysis of five HLA SNPs.

Haplotype analysis was performed between HBV patients and control group and two blocks were produced. The first block (block 1) consists of the two HLA-DQ SNPs rs2856718 and rs9275572, while, the other block (block 2) was the HLA-DP SNPs rs3077 and rs9277535 [Figure 1a and b]. Three out of the four haplotypes in block 1 were found to be significant [Table 6]. The haplotype AG which included the risk allele for rs2856718 was found to be significant with (p = 0.0366 and freq. = 0.416); the haplotype GA, which includes the protective alleles, was found to be significant with a p value<0.0001 and a frequency of 0.259 within the population. In addition, the haplotype AA which included the risk allele for rs2856718 and the protective allele for rs9275572 was also found to be significant with (p = 0.0284 and freq. = 0.098). For block 2, only one haplotype GG was found to be significant with (p = 0.0121 and freq. = 0.147).

**Table 6:** Haplotype analysis for HLA gene polymorphisms between patients (Group I+II+III+IV) and controls.

Haplotype		Freq.	Case, Control Ratio Counts	Case, Control Frequencies	Chi Square	P Value
**Block 1**						
**rs2856718**	**rs9275572**					
A	G	0.416	677.6∶888.4, 466.1∶719.9	0.433, 0.393	4.371	**0.0366**
G	A	0.259	349.9∶1216.1, 364.0∶822.0	0.223, 0.307	24.456	**7.60×10^−7^**
G	G	0.227	367.7∶1198.3, 256.4∶929.6	0.235, 0.216	1.34	0.247
A	A	0.098	170.8∶1395.2, 99.6∶1086.4	0.109, 0.084	4.805	**0.0284**
**Block 2**						
**rs3077**	**rs9277535**					
A	A	0.638	977.4∶588.6, 779.9∶408.1	0.624, 0.657	3.065	0.0800
G	G	0.147	254.0∶1312.0, 152.1∶1035.9	0.162, 0.128	6.294	**0.0121**
A	G	0.109	169.6∶1396.4, 130.8∶1057.2	0.108, 0.110	0.022	0.8816
G	A	0.105	165.0∶1401.0, 125.2∶1062.8	0.105, 0.105	0	0.9955

Similarly, haplotype analysis was performed between HBV patients and HBV-cleared individuals, which included the two HLA-DQ SNPs rs2856718 and rs9275572. Three out of four haplotypes were found to be significant [Table 7]. The haplotype AG which included the risk allele for rs2856718 was observed more frequently with a freq. = 0.414 and was found to be significant with a p value = 0.0139, while the haplotype GA which included the risk allele for rs9275572 was also highly significant (p<0.0001 and freq. = 0.245). The haplotype which included the risk alleles for both the SNP was also significant with a p = 0.0297 but its frequency was only 1%.

**Table 7:** Haplotype analysis for HLA gene polymorphisms between patients (Group I+II+III+IV) and clearance group.

rs2856718	rs9275572	Freq.	Case, Control Ratio Counts	Case, Control Frequencies	Chi Square	P Value
A	G	0.414	673.2∶892.8, 225.4∶380.6	0.430, 0.372	6.053	**0.0139**
G	A	0.245	345.2∶1220.8, 187.3∶418.7	0.220, 0.309	18.577	**1.63×10^−5^**
G	G	0.238	372.3∶1193.7, 144.6∶461.4	0.238, 0.239	0.002	0.9649
A	A	0.103	175.3∶1390.7, 48.7∶557.3	0.112, 0.080	4.728	**0.0297**

## Discussion

In this study, four SNPs (rs2856718, rs3077, rs9277535 and rs9275572) belonging to the HLA-DP and HLA-DQ region were found to be significantly associated with HBV susceptibility. The SNP rs2856718 which is located in the intergenic region between HLA-DQA2 and HLA-DQB1 was found to be dominantly associated with HBV infection, which was consistent with the findings of a previous study [14]. For rs2856718, the risk genotype, AA, was found to be more predominant among HBV-infected patients (freq. = 0.40) when compared to HBV-cleared subjects (freq. = 0.15). Under the dominant model, this study revealed that the non-risk allele G was found to be strongly associated with HBV clearance when compared to AA genotype carriers. This suggests that inheriting a single rs2856718-G allele would reduce the risk of an individual to progress into chronic HBV infection.

Similar observations were made for the non-risk allele G of rs7453920, which was also dominantly associated with HBV clearance. In agreement with the observations of Hu et al. [18], no significant association was observed with liver cirrhosis/HCC risk. The SNP rs7453920 that belongs to intron 1 of HLA-DQB2 region failed to show any association with susceptibility to HBV infection as observed in the study of Mbarek et al. [14].

The SNPs, rs3077 and rs9277535 have been analyzed in several studies [13], [14], [15], [16], [17], [18], [19], [21], [22]. In the present study, the rs3077-G allele (freq. = 0.23) was found to be the minor allele compared to the A allele (freq. = 0.77) among healthy uninfected subjects. This finding is consistent with the observations from cohorts with different ancestries such as Caucasian (G = 0.16), Mexican in California (G = 0.21), Tuscan in Italy (G = 0.22) and Maasai in Kenya (G = 0.30), who were included in the HapMap project and from the pilot studies of 1000 Genomes project conducted among Caucasian population (G = 0.13) (http://www.ncbi.nlm.nih.gov/projects/SNP/snp_ref.cgi?rs=3077). A similar observation was made by Vermehren et al. [23], with a G allele frequency of 0.18 among healthy Caucasians. The study further reported that the rs3077-G was recessively associated with the risk to HBV infection. This was in agreement with our study where the SNP rs3077 was found to be significantly associated with HBV infection, predominantly among HBV patients who carried the homozygous GG genotype. Surprisingly, our observations differed from other Asian studies in terms of allelic distributions. Similar work described the same findings with the major G allele being a risk for HBV infection [19]. Other studies conducted on Chinese [15], [16], [17], [22], Japanese [13], [24], Thai [13] and Korean [24] subjects reported that the minor A allele was protective against HBV infection. A phylogenetic analysis using four VNTRs and one STR revealed that Japanese, Chinese (Han, Hui and Uygur populations) and Kazakhs formed one cluster while two European populations (Greek and Italians) formed another cluster with the Saudi Arabian population sample, suggesting that the Saudi Arabian population might be more closely related to the Caucasian [25]. That might be one reason for this difference in genotype distribution. In addition, no significant association was observed for rs3077-G in relation to HBV viral clearance but a significant association was estimated in the case of persistent HBV infection under the dominant model but in the opposite direction, suggesting that the AA genotype may play a role in the progression of HBV infection to chronicity, while the G allele might be protective against the progression. This could be supported with the findings of Tseng et al. [26] who reported that the GA and GG haplotypes of rs3077 and rs9277535 were associated with a higher HBeAg seroconversion rate among chronic HBV patients undergoing PEG-IFN therapy. However, no association was observed with progression to liver cirrhosis or HCC in this study, which is in agreement with An et al. [16] but in contradiction with the conclusions of Hu et al. [18].

The SNP rs9277535 was also found to be significantly associated with HBV susceptibility. The GG genotype was more prevalent among HBV patients (GG = 0.09) than controls (GG = 0.05) and was thus found to be recessively associated with HBV risk. This was consistent with studies conducted on different Chinese populations that reported the G allele to be susceptible to chronic HBV infection [17], [19], and the A allele to have a protective effect against HBV [16], [17], [21], while others reported a significant association with HBV clearance [15], [21], [22]. Other studies have reported no significant association with HBV infection or its recovery among Caucasians [23] and among African- and European-Americans [27]. Certain discrepancies regarding the distribution of alleles as observed for rs3077 [15], [16], [17], [18], [19], [21], [22] remain, but the observations of this study (G = 24%) were comparable to the data published for Yoruban cohorts in the HapMap project and in the 1000 Genomes project, with the minor G allele frequency varying between 10.3–12%. Furthermore, we found no association for rs9277535 with HCC risk which was consistent with the results of Hu et al. [18].

In addition to the above four SNPs, we analyzed a novel HLA-DQ SNP rs9275572-A, which seemed to show a protective effect against HBV infection and also showed a significant association with HBV clearance. This SNP has been reported to have a significant association with HCV-induced HCC among Japanese [28]. However, in this study no significant association was observed with HBV-related liver cirrhosis or HCC, and a similar result was observed in the study conducted by Li et al. [29].

The haplotype that included the risk alleles of the two SNPs rs3077 and rs9277535 was found to be significantly associated with HBV susceptibility. This can be substantiated by the evidence from a study that reported these SNPs to be strongly associated with the regulation of mRNA expression of HLA-DPA1 and -DPB1, by lowering their expression with the increasing risk of chronic HBV [30].

In summary, this study demonstrated that the genetic variations in the HLA-DP and -DQ genes are strongly associated with HBV susceptibility among Saudi Arabian population. Furthermore, a major finding of this study is that SNPs that belong to HLA-DQ variants are linked to HBV viral clearance.
